# Reference Values for Cardiopulmonary Exercise Test in Children—How to Report Them Correctly?

**DOI:** 10.3390/jcm14227989

**Published:** 2025-11-11

**Authors:** Przemysław Kasiak

**Affiliations:** 3rd Department of Internal Medicine and Cardiology, Medical University of Warsaw, 61 Żwirki i Wigury Street, 02-091 Warsaw, Poland; przemyslaw.kasiak@wum.edu.pl

**Keywords:** CPET, pediatric athlete, reference values, children, normative data, cardiopulmonary exercise test

## Abstract

Cardiopulmonary exercise testing (CPET) is a gold standard to assess cardiorespiratory fitness (CRF). CRF varied through the lifespan, increasing in children until early adulthood and then gradually declining. Reference values for CPET are used to check whether the child’s CRF falls within the normal range. The differences between directly measured and normative age-adjusted exercise data may suggest pathology and are helpful during the diagnostic process. Deriving reference values for children is particularly challenging. Moreover, many children participate in sports, some at an advanced/elite level, which translates into specific adaptations in CPET. The ATS/ACCP statement on CPET presented a checklist that should be followed when reporting reference values. However, the checklist originally focused on adults. This aggravates the quality of reporting pediatric reference values for CPET, making between-studies comparisons difficult. This review (1) presents a step-by-step protocol to fulfill all requirements from the ATS/ACCP statement in the pediatric population, and (2) summarizes the key challenges in deriving reference values for CPET in children, especially among pediatric athletes. Additional recommendations to enrich the quality of reporting reference values for CPET in pediatric athletes were also discussed.

## 1. Introduction

Cardiopulmonary exercise test (CPET) allows a comprehensive evaluation of response to exercise and cardiorespiratory fitness (CRF) [1,2]. There are several indications for CPET, including dyspnea of unknown etiology, exercise intolerance, suspected congenital heart defect, non-severe functional symptoms, or even a fitness check-up [1,3,4]. CRF is a strong predictor of health outcomes across the lifespan [5]. Higher CRF in childhood is associated with a lower risk of cardiovascular diseases (CVD) in adulthood [6]. Although there are several methods to assess CRF, CPET is described as the most informative and cost-effective test [2]. The key parameter indicating CRF is maximal oxygen uptake (VO_2max_) [7]. VO_2max_ merges the circulatory, respiratory, and muscular systems [8]. Together with other CPET variables, they present a comprehensive picture of CRF [9].

Normative data for CPET provide comparative ranges to evaluate whether the achieved CPET results could indicate pathology or are a variant of normality [10]. Several reference values have been developed for CPET [11], and most of them were for adults. There are also reference values for children, but usually developed from smaller populations [12,13,14]. Preferably, reference values are derived from wide registries, as the Fitness Registry and the Importance of Exercise National Database (FRIEND), to ensure representativeness of the population [9].

Normative data and the protocol for conducting CPET change throughout life [11,15]. VO_2max_ increases until early adulthood, after which it begins to gradually decline for about 6–8 mL/kg/min per decade [16]. There are specific methodological issues that should be considered exclusively for children or adults when conducting CPET [17], especially equipment and criteria for maximum effort, should be adjusted in pediatric participants [15].

Pediatric cardiology and the number of studies on pediatric CPET are increasing [18,19]. To enable a reliable assessment of the quality of research, specific areas must be addressed and critically assessed. Reference values for CPET should be reported according to the American Thoracic Society (ATS) and the American College of Chest Physicians (ACCP) statement [20]. The statement presents a 14-point checklist that summarizes the key methodological issues. Typically, 0–6 indicates study of low quality, 7–9 of moderate quality, and 10–14 of high quality [12,14]. Other guides on CPET have been developed; however, they do not present such a unified tool to stratify quality [2]. Despite this statement being published over 20 years ago, it has not been updated. The checklist originally focused on adults’ CPET and was adapted to the pediatric reference values in different ways [20]. This led to a highly heterogeneous methodology [13].

Therefore, the primary aim of this review was to present a step-by-step protocol on how to address all points from the ATS/ACCP statement to the pediatric normative data for CPET, including athletes. The secondary aims were to highlight the key challenges in deriving the reference values for CPET in children, with special considerations for pediatric athletes, and to discuss additional recommendations beyond the ATS/ACCP statement on how to enrich the quality of reference values for CPET in pediatric athletes.

## 2. Materials and Methods

This review presents a narrative framework that combines clinical and experimental studies. The searching strategy focused on the guidelines/clinical consensus statements and the latest articles. All the articles that were broadly relevant to pediatric exercise science and normative data for CPET were considered. Recommendations for pediatric athletes were proposed based on the guidelines from the European Society of Cardiology, American Heart Association, and American College of Cardiology. The most prominent studies were selected to illustrate key challenges for reference values in children and adaptations of qualitative assessment for pediatric CPET.

## 3. Challenges in Deriving Reference Values for CPET in Children

The young developing individuals are a unique population [19]. Children are more difficult to motivate for maximum CPET [21]. Other alternative methods for estimating CRF, such as Progressive Aerobic Cardiovascular Endurance Run (PACER), field testing, or 20 m shuttle run, are more common than in adults [22]. However, no method can completely replace CPET with the same value and accuracy, and all those methods are only a surrogate [1].

The growth and development body proceed dynamically, but often at a different pace [23]. Sometimes, two adolescents, despite similar chronological age, have significantly different exercise results due to different onsets of puberty [24]. It could be due to changes in lean body mass, cardiac output, and chest dimensions [25]. Absolute data is not always accurate, and scaling methods are necessary [26]. There is still no clear age cutoff to start CPET in children, but those under 6–7 years old often demonstrate limited compliance [17].

Drawing a clear line between a pediatric athlete, the exerciser, and just an untrained individual is a difficult task [27,28]. There are attempts to define who is a pediatric athlete and to set clear criteria for the advancement level [29]. Children often participate in sports during extracurricular activities, while adolescents train in sports clubs [19]. Some pediatric athletes also participate in sports competitively. It is common to participate in professional competitions and play against adult athletes (e.g., in soccer, where professional careers begin around the age of 15–16) [30].

Children also suffer from cardiovascular diseases (CVD) and injuries, with inherited conditions and acute musculoskeletal disorders being the most common [31]. In the worst-case scenario, it leads to sudden cardiac arrest (SCA) [32]. Although SCA is rare among children and has a frequency comparable to the adult population, there is a social fear of SCA in young individuals [19]. CPET, in accordance with properly selected reference values, may be useful in the pre-participation screening for athletes or during the diagnosis of symptomatic individuals, and finally translates into a reduction in the frequency of SCA during exercises and CVD in general [32].

To properly assess a pediatric patient, normative data should suit demographic characteristics and testing protocol [4,33]. The same best-fitting set of reference values should be selected and consistently used for an individual [15]. Even in untrained children, specific electrocardiographic (ECG) or echocardiographic changes may occur compared to adults, e.g., sinus bradycardia [34]. In adults, some ECG changes may be a normal adaptation to exercise in athletes and pathology in untrained individuals simultaneously [35]. These include incomplete right bundle branch block or ectopic atrial or junctional rhythm [10]. Moreover, several characteristic echocardiographic changes among athletes occur, including pediatric athletes. Elite pediatric athletes develop cardiac remodeling to a lesser extent than adult athletes in response to exercise [36]. Reference values for CPET in young adult athletes also differ from those in untrained individuals [4]. Athletes may observe excessively elevated ventilatory efficiency slope, which in untrained individuals may indicate heart failure [37].

To sum up, key challenges in deriving reference values for CPET in children were presented in Table 1. Several premises emerge that cardiovascular physiology is different in children compared to adults or between athletes and untrained subjects. This highlights the need to discuss the development and quality assessment of reference values in pediatric participants.

## 4. Adapting ATS/ACCP Statement to the Pediatric Population

The quality assessment consists of 14 items [20]. This is the only available checklist that allows for the critical assessment of the quality of research on CPET and is used in systematic reviews or pooled analyses [12,13,14]. If the condition is met, 1 point is awarded; if it is not met or omitted, 0 points are awarded.

Given the differences between adult and pediatric CPET, some items may be incorrectly classified as not met (e.g., specific criteria of maximum effort). Moreover, other items are often neglected and not reported because they potentially seem to be inappropriate for the pediatric population (e.g., exclusion of smokers). Misclassification of items may underestimate or overestimate the final quality score of a study and translate into incorrect results (e.g., during sensitivity analysis in meta-analysis).

**Figure 1:**
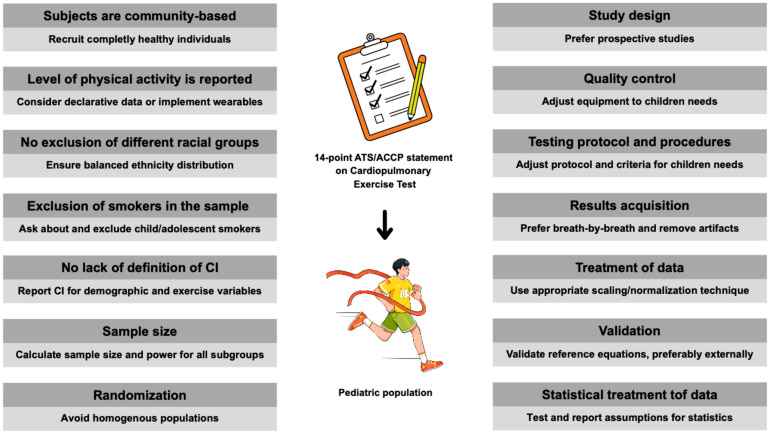
Points to consider when applying the ATS/ACCP statement in children’s CPET. Abbreviations: ATS, American Thoracic Society; ACCP, American College of Chest Physicians; CI, confidence intervals.

Below, each point was discussed for the needs of the pediatric and adolescent populations, especially considering athletes. The proposed adjustments are complementary to the original principles of the ATS/ACCP statement. The adjusted view was presented on how to go step-by-step to properly address all criteria in pediatric reference values for CPET. It is intended as a manual for researchers in reporting and classifying studies or clinicians for selecting the best-matching normative data for their patients. A visual summary of the key considerations from the ATS/ACCP statement in children is presented in Figure 1.

### 4.1. Subjects Are Community-Based

Some studies include participants who have not been diagnosed and are healthy at the time of CPET, but undergo exercise testing during the diagnostic process [3,38]. No study has yet presented data on how such a diagnostic process ended and whether the population would ultimately be classified as clinical. If the participants are hospital-based a data regarding the final diagnosis would be necessary. The CHEER registry presented a different approach. About two-thirds of the participants underwent CPET due to clinical evaluation [4,39]. Of these participants, only individuals with no heart or lung disease were included to avoid factors adversely affecting CRF. Two alternative approaches appear here: classifying all into reference data development and providing the percentage of diagnosed, or including only the undiagnosed.

In studies of pediatric athletes, where CRF is of particular importance, standard pre-participation screening could be conducted priorly to exercise testing [40]. Although pre-participation screening guidelines vary significantly, those appropriate for the location could be used [19]. Both will ensure that investigators are confident that their participants/athletes were healthy. Then any findings could be considered a variant of normality, not pathological. This practice is often used when presenting reference data for the interpretation of echocardiograms or ECGs [10]. However, it has not been implemented routinely in the derivation of CPET in children yet. Normative values developed in this way consider physiological adaptations during growth and/or exercise [37]. In summary, when recruiting the study participants, their medical evaluation should be conducted to exclude disturbing factors and classify subjects as community-based.

### 4.2. Level of Physical Activity Is Reported

Physical activity levels are particularly important in pediatric research, especially for athletes [28]. It would be minimally achieved by an additional question about physical activity in the participant selection criteria. However, this would lead to some bias and reliance on subjective, declarative data from children or parents. Records from wearable devices, training diaries, or physical education lesson plans will be more reliable [41].

Most children will have some level of physical activity—at school, in a sports club, or while playing with peers [42]. Any information regarding habitual physical activity will be helpful and allow for a further randomization process [28]. However, to avoid overrepresentation of less active participants (those who declare themselves exercisers), the motivations and intentions of physical activity should be defined [43]. During the recruitment process, motivation for physical activity may be evaluated—whether it is solely to improve health and well-being or to enhance performance for competitive purposes [44]. The weekly amount of physical activity or training regimen could be addressed in the table or paragraph about the demographic characteristics.

### 4.3. No Exclusion of Different Racial Groups

There are differences in CRF between races [45]. This is particularly important in studies on athletes, as in elite sports, top academies and colleges recruit players from all over the world [46]. Similarly, as globalization and traveling progress, the local ethnicities are mixed, and this could influence the ethnic distribution in study populations [47]. Ethnicity could be guessed based on the study location (e.g., normative data from Europe has been derived from White, while from China, from Asians). However, this has a key shortcoming, as different ethnicities currently live all over the world and could be recruited as study participants [48]. The number of participants from each ethnicity should be reported, and if data for ethnicity are unavailable, this should be stated as a limitation.

### 4.4. Exclusion of Smokers in the Sample Studied

Tobacco smoking is not uncommon among children, especially adolescents [49]. Typically, the reference values will also include adolescents [26]. School and social environments often promote early nicotine initiation [50]. The development of alternative products, such as heated tobacco products or e-cigarettes, leads to strong differences in local legislation and prohibition [51]. Although the exclusion of smokers may seem to apply only to adults, it should also be clearly reported in children, especially adolescents approaching adulthood [50].

### 4.5. No Lack of Definition of the Confidence Limits for Individual or Specified Characteristics

Usually, only predicted values are provided (e.g., estimated VO_2max_) without declaring ranges within these predictions may fluctuate. It is more important in children than in adults because their body dimensions change dynamically [52]. Anthropometric changes (weight, height, chest dimensions, etc.) affect CRF [53]. Preferably, reference equations should be developed besides tabulated data [26]. Several studies present various forms to normalize CPET data (e.g., linear regression or allometric models) [13,14]. In addition to reference equations, their accuracy measures and assumptions must be outlined [54], including confidence intervals for predictions. This allows an assessment of whether the actual result from CPET, compared to the predicted one, is within the norm (i.e., within the confidence interval) or not [55].

### 4.6. Sample Size

An appropriate sample size is required to support the representativeness of the study population and ensure power for statistical analyses. There are different methods to establish and achieve the required sample size [56]. For prospective studies, it is crucial to consider the size of similar studies in the past and establish the minimum required sample [57]. Post hoc testing of the statistical power is mandatory for retrospective studies. Several easily accessible software packages facilitate power analysis, such as G*Power [58]. It is important to report the power of all subgroups for which reference values will be developed (e.g., boys, girls, age subgroups, VO_2max_ percentiles, etc.). Current studies selectively report sample data are provided for the entire study population, while reference values are developed for smaller subgroups (whose power is not reported). This makes it difficult to assess the reliability of the presented reference values.

### 4.7. Randomization

Reference values for CPET usually relied on the convenience sampling method [14,59]. Random sampling leads to a more representative and universal representation of the data [60]. If the data were derived from a homogenous sample (e.g., elite athletes), the reference values will be systematically overestimated and relevant only for a narrow population [9]. It is not bad in itself if this is the aim of the investigators. However, physical activity levels can vary significantly between populations, and in some societies, children have significantly higher levels of non-exercise physical activity than in others [61]. The level of basic habituated physical activity also differs between cultures [62]. This may be due to differences in school curricula between countries, which include more physical education lessons [63,64]. In studies of pediatric athletes, it is important to ensure equal participation of elite and amateur/recreational athletes [29]. Among children, and especially pediatric athletes, the randomization for deriving the CPET normative data will most often refer to the level of physical activity and performance caliber [65]. Therefore, the randomization process should be conducted to mirror the target population for which the normative values aim to be applied (this may be the general population, athletic, or clinical).

### 4.8. Study Design

Preferably, studies deriving reference values for CPET should be prospective [3]. Sometimes it is impossible to conduct a prospective study that recruits participants long enough to achieve the required sample size, and studies presenting normative values are often retrospective [38]. A common practice is combining databases from different studies [66]. If such a merging protocol is used, the data should be corrected and/or reduced to include uniform inclusion and exclusion criteria. All elements of a properly composed study design also include defining the target population (both biological and chronological age, degree of maturity [67]), inclusion and exclusion criteria (pediatric-specific criteria for maximum effort in CPET [17]), and defining the protocol and equipment (type of ergometers [14]). A priori criteria should be defined to recruit participants in large clinical centers and hospitals, from which normative values could potentially be developed in the future.

### 4.9. Quality Control

Assessment of data quality is often lacking. It is common practice to refer to other publications/guidelines/position papers without explicitly explaining whether quality assessment was used. Quality control includes several procedures that must be adapted to the pediatric population. Children have smaller face sizes than adults; therefore, face masks for CPET should be appropriately selected to avoid free airflow [15]. Children are also more difficult to motivate to perform the maximum CPET than adults [17]. Staff must ensure that the pediatric participant understands the protocol and is aware of the coming exertion [68]. Special attention should be paid to thoroughly explaining and familiarizing the child with the CPET protocol in a friendly manner [69]. Considering specific areas where children differ from adults in quality control procedures will ensure that the data is reliable and accurate.

### 4.10. Exercise Testing Protocol and Procedures

ATS/ACCP guidelines precisely described how to report CPET. However, some points should be especially considered. Children, especially those under 6–7 years of age, often do not cooperate during CPET [17]. It may be helpful to have a parent present during the test to make the child feel safe. Different protocols are used for CPET; however, not all of them are appropriate for children (e.g., the Bruce protocol for treadmill is more appropriate for adults) [17].

It is necessary to report modality-specific norms. Direct cross-modality comparisons should be made with caution. Typically, the use of a cycle ergometer allows more accurate measurements (e.g., stress ECG) than a treadmill due to the more stable position [70]. However, CPET results are higher on the treadmill for about 7–13% and transferring normative data from treadmill to cycle CPET could lead to erroneous underestimation [71]. When conducting CPET in children, smaller body dimensions should be considered, and the size of the ergometers should also be adjusted [17].

There is no clear consensus on which criteria of maximal effort are most tailored for the pediatric population [72]. Pediatric research often uses an RER > 1.1, which is one of the main criteria for assessing maximal exertion in adults [15]. RER could be lower (~1.0), and the VO_2_ plateau does not always occur in children, despite performing maximal effort [73]. Typically, a cutoff point for age-predicted maximum heart rate is used (e.g., 95% or 80%). This is problematic because maximum heart rate is often highly variable between individuals, and setting one arbitrary cutoff can lead to over- or under-classification of effort as maximal [74]. Some studies have even used blood lactate levels to confirm maximal CPET [75]. Considering pediatric CPET, the focus may be even more on volitional exhaustion and confirmation of maximal effort by a qualified supervising physiologist [76]. Hence, when analyzing the quality of the CPET protocol, exercise criteria that are stated clearly and even consider some selected items can still be adequate. It should be emphasized that it is a combination of several factors that leads to truly maximum CPET, rather than choosing one.

### 4.11. Results Are Obtained by Breath-by-Breath Analysis or Mixing Chamber and Treated in Accordance with ATS/ACCP Guidelines Statement

Both breath-by-breath and mixing chambers can be used in the pediatric population; however, breath-by-breath is preferred [17]. Tidal volumes in children are lower, and respiratory rates are higher [77]. The ATS/ACCP statement recommends 30–60 s averaging intervals [20]. Shorter averaging intervals can be used in children than in adults (e.g., 20 s). It is also worth considering reporting data for children from multiple intervals (10–20 s vs. 30–60 s) [15]. Due to lower compliance among children, special attention should be paid to the removal of artifacts [78]. Artifacts may appear during speech (questions about CPET protocol) or coughing (if the patient is symptomatic) [78]. Artifacts can be excluded by considering the depth of respiration, including tidal volume < 50% of the median for a given session [17]. The terminal data should be calculated from the averaged interval (VO_2max_), and not from the highest peak value (VO_2peak_) [17].

### 4.12. Treatment of Data

**Figure 2:**
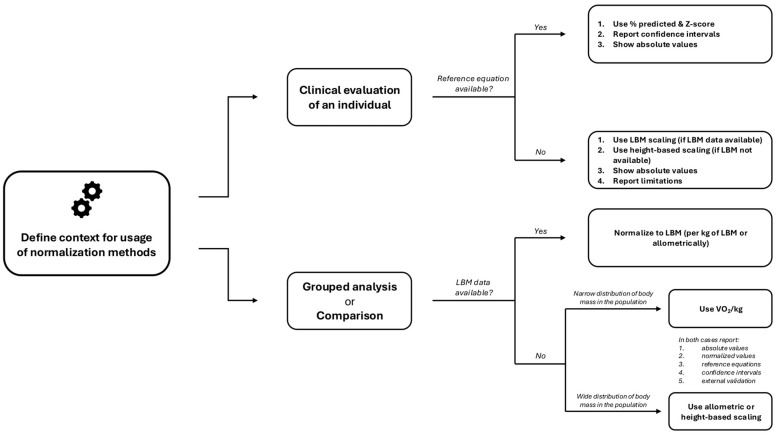
Guide to choose the normalization methods according to the context and available data for pediatric CPET. Abbreviations: LBM, lean body mass; VO_2_, oxygen uptake.

Treatment of data in children is especially challenging [79]. Data should be reported in an adjusted manner (for body dimensions or lean body mass). There is a set of normalization methods to consider. When investigating broad populations with wide age and anthropometric distributions, it may be worthwhile to use allometric normalization [55]. Within-subject investigation using the intraclass correlation coefficient can also be used [80]. This allows for comparison of whether measurements carried out in different periods (often seen in cohort studies) are consistent over time [81]. The treatment of data is especially challenging in children [17], and reference values for CPET should not report a one “normal range”. In pediatric research, the Z-score is well-suited for continuous variables monitored in CPET. Additional normalized data supported by confidence intervals will allow clinicians to assess the degree of deviation among their patients [79]. Figure 2 presents a guide to choosing the normalization methods according to the context and available data for pediatric CPET.

### 4.13. Validation

Validation is a necessary procedure to test the transferability of the developed reference equations [37]. The ATS/ACCP statement recommends external validation [20,82]; however, this requires recruiting additional participants and is often neglected. There are various statistical methods used for validation: comparison of the directly measured and predicted values (e.g., by Student’s *t*-test or Mann–Whitney U-test), univariate linear regression of measured values against predicted values, or simple calculation of absolute errors [82]. There are other validation methods to consider, such as cross-validation or bootstrapping [83]. Demographic data of validation and derivation cohorts should be reported separately and controlled for confounding factors [83,84].

### 4.14. Statistical Treatment of Data

As previously underscored, in children, normative values for CPET are normalized. Adjustments are made using a variety of statistical methods. To allow a clear assessment of whether the appropriate statistical method was used, assumptions should be reported. Each statistical method has certain assumptions that justify its use: for linear regression, there are autocorrelation, homoscedasticity, and independence of observations [54], while for the Student *t*-test, data must follow a parametric distribution [85]. The assumptions do not necessarily need to be reported in the manuscript directly, but at least they should be noted that such assumptions were tested (in the paragraph about statistical analysis). Preferably, they could be included in a supplementary material.

## 5. Discussion

The adaptation of the checklist from the ATS/ACCP statement to the pediatric population was presented. Key takeaways are: (1) specific differences between the pediatric and adult populations should be considered when reporting the protocol or assessing the quality of studies, (2) the original framework of the ATS/ACCP statement remains unchanged and key items should be evaluated in both populations and (3) some additional areas beyond the original checklist may be addressed in pediatric studies. This is the first review to discuss the available methods of quality assessment for CPET in pediatric research. As the number of reference datasets in children increases, there is a need to systematize the reporting methodology and follow a unified scheme [13,14].

### 5.1. Further Perspectives for Reference Values in Pediatric Athletes

The description of the study population may address an assessment of maturation and biological age. Both strongly influence CRF [86]. The impact of puberty on CRF has led some sports associations to introduce stratification into early- and late-maturing children [87]. Late-maturing children may train and compete with children of younger chronological age but comparable biological age [87]. Given the growing importance of a comprehensive assessment of the pediatric athlete, such an issue should be clearly reported.

Studies of pediatric athletes should accurately identify the effective information on the level of training [27]. It is common for children to be described as “athletes” without additional insights [28]. This leads to ambiguity and overclassification. A unified method of recruiting participants, confirmed by a sports club ID card, academic sports association membership, would ensure credibility [88]. Determining weekly training hours (e.g., by wearables, app, or diary) and competition level, and using the type of sport according to the European Society of Cardiology (skill, power, mixed, endurance) classification, would be helpful [89]. In children, family history is more important and a stronger risk factor for several diseases than in adults [90]. In parallel with assessing current health status during CPET (symptomatic/hospital-based or asymptomatic/community-based), family history and medical records should be outlined.

**Figure 3:**
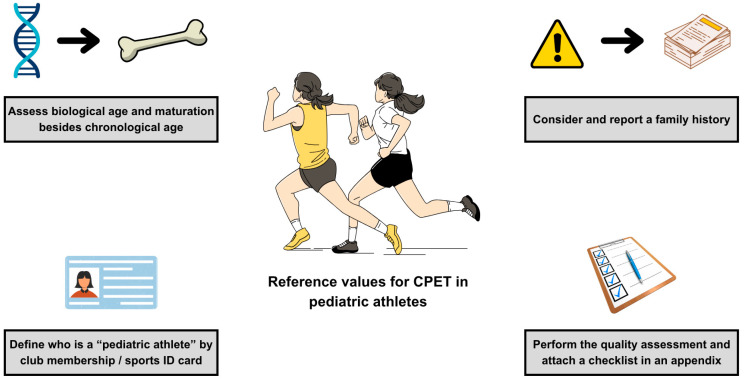
Recommendations for tailored reference values for pediatric athletes. Abbreviations: CPET, cardiopulmonary exercise test.

Few studies explicitly refer to the ATS/ACCP statement and present quality assessment of their studies. Such “good practice” was presented by Gavotto et al. [3] and Amedro et al. [65]. It is often difficult to classify some unclear statements made by the authors in their articles. This led to interrater variability and subjective interpretation of unclear statements. An excellent solution would be to present a qualitative assessment in the form of supplementary material/appendix solely by the authors, along with the main article (similar to the STROBE or CONSORT checklists) [91]. Further recommendations for tailored reference values for pediatric athletes are presented in Figure 3.

### 5.2. Illustrative Scoring of Reference Values

Exemplary application of ATS/ACCP guidelines to the existing dataset has been presented in a study by Gavotto et al. [3]. Table 2 presents illustrative scoring with evaluation notes and clinical implications, constituting a manual for qualitative assessment of pediatric CPET. The justification for why individual recommendations were awarded points or not, with an exact claim from the manuscript, was also described.

This study finally received 11 points. This allows comparison of facts in the article with direct points from the checklist. Even when a particular quality point has not been directly stated in the article, the checklist in the appendix completely dispels any doubts (e.g., information about the exclusion of smokers or regarding the averaging interval).

### 5.3. Limitations

Despite key concepts being underscored, at some points, a broader evidence synthesis is required. There was no formal study selection or risk of bias assessment. The selection of studies was not carried out with predefined inclusion criteria. The main objective was to highlight prominent findings in the reliable reporting of pediatric CPET. The most illustrative and impactful studies have been discussed narratively. Therefore, this review does not provide a data synthesis of all current datasets of reference values but serves as a methodological manual.

## 6. Conclusions

Key points from the ATS/ACCP statement for assessing pediatric reference values for CPET were summarized. The manual on their implementation in pediatric research was presented, especially considering athletes. The ATS/ACCP checklist can be used in adults and in children to evaluate their risk of bias after considering the specificity of both. Additional insight may be addressed exclusively in the pediatric CPET to enrich its quality.

## Figures and Tables

**Table 1:** Key challenges in deriving reference values for CPET in children.

Challenge	Proposed Solution
Worse motivation and compliance in children during CPET, especially those 6–7 years old	Ensure verbal encouragement during CPET
Rapid growth and changes in body dimensions (including weight, height, fat-free mass, chest dimensions, lung capacity)	Implement appropriate scaling and normalization techniques
Varied definitions of “healthy” participant (symptomatic vs. asymptomatic) or pediatric athlete	Clearly report medical history of participants and ensure a follow-up when recruiting symptomatic children
Discrepancies between chronological age and biological age within-participant and between-participants	Report biological age during recruitment
Difficulty in confirming the maximum exertion, which often requires a qualified supervising physiologist	Consider a set of criteria for confirmation of maximal effort
The need to use smaller, less available equipment in pediatric sizes	Provide equipment adjusted for pediatric populations
Parental/guardian consent and child’s assent when appropriate	Obtain all necessary documents according to local law/legislation
Recruitment of qualified personnel able to communicate with a pediatric patient	Train personnel on how to communicate with a child and ensure a friendly atmosphere
No clear definition of minimal accepted age to derive reference values for CPET	Reconsider whether the child is cooperating (if not, choose a different method to assess fitness)

Abbreviations: CPET, cardiopulmonary exercise test.

**Table 2:** Illustrative scoring for a set of normative data for pediatric CPET based on ATS/ACCP recommendations.

Item	Proposed Score	Evaluation Note/Clinical Implication
Subjects are community-based	No	Participants were referred for CPET due to clearly listed symptoms
Level of physical activity is reported	No	There was no method (diary, questionnaire, etc.) to assess the level of physical activity
No exclusion of different racial groups	Yes	All racial groups were considered, and ethnicity has not been listed among the exclusion criteria
Exclusion of smokers in the sample studied	Yes	Smokers were not considered *
No lack of definition of the confidence limits for individual or specified characteristics	Yes	Z-scores and limits of normal were reported
Sample size	Yes	The study sample consisted of 909 participants with a separate validation cohort. There was also an equal sex distribution and inclusion of overweight/obesity children.
Randomization	No	There were no procedures to avoid overrepresentation of one particular group (e.g., more physically active children)
Study design	Yes	The study population came from prospective controlled studies
Quality control	Yes	Several quality control procedures were applied, including the same equipment in all laboratories
Exercise testing protocol and procedures	Yes	Full protocol described in detail in a separate paragraph “CPET procedures”
Results are obtained by breath-by-breath analysis or mixing chamber and treated in accordance with ATS/ACCP guidelines statement	Yes	Breath-by-breath method is directly stated in a paragraph “CPET procedures”
Treatment of data	Yes	Data were averaged to avoid noise variables *
Validation	Yes	There was an external validation
Statistical treatment of data	Yes	The procedures to construct the best-fitting Z-score models have been described

Note: * only stated in the checklist, but not in the main article. Abbreviations: ATS/ACCP, American Thoracic Society/American College of Chest Physicians; CPET, cardiopulmonary exercise test.

## Data Availability

Not applicable.
